# Optical, Electronic Properties and Anisotropy in Mechanical Properties of “X” Type Carbon Allotropes

**DOI:** 10.3390/ma13092079

**Published:** 2020-05-01

**Authors:** Jiao Cheng, Qidong Zhang

**Affiliations:** 1 Xi’an University of Architecture and Technology, Xi’an 710055, China; chengjxatu@163.com; 2 School of Microelectronics, Xidian University, Xi’an 710071, China

**Keywords:** carbon allotropes, electronic properties, anisotropic properties, optical properties

## Abstract

Based on first-principle calculations, the mechanical anisotropy and the electronic and optical properties of seven kinds of carbon materials are investigated in this work. These seven materials have similar structures: they all have X-type structures, with carbon atoms or carbon clusters at the center and stacking towards the space. A calculation of anisotropy shows that the order of elastic anisotropy in terms of the shear modulus, Young’s modulus and Poisson’s ratio of these seven carbon materials with similar structure is diamond < supercubane < T carbon < Y carbon < TY carbon < cubane-diyne < cubane-yne. As these seven carbon materials exhibit cubic symmetry, Young’s modulus has the same anisotropy in some major planes, so the order of elastic anisotropy in the Young’s modulus of these seven main planes is (111) plane < (001) plane = (010) plane = (100) plane < (011) plane = (110) plane = (101) plane. It is also due to the fact that their crystal structure has cubic symmetry that the elastic anisotropy in the shear modulus and the Poisson’s ratio of these seven carbon materials on the seven major planes are the same. Among the three propagation directions of [100], [110], and [111], the [110] propagation direction’s anisotropic ratio of the sound velocity of TY carbon is the largest, while the anisotropic ratio of the sound velocity of cubane-diyne on the [100] propagation direction is the smallest. In addition, not surprisingly, the diamond has the largest Debye temperature, while the TY carbon has the smallest Debye temperature. Finally, TY carbon, T carbon and cubane-diyne are also potential semiconductor materials for photoelectric applications owing to their higher or similar absorption coefficients to GaAs in the visible region.

## 1. Introduction

The existing materials generally have some deficiencies in performance, for example, the indirect band gap of diamond-Si limits its application in photoelectric devices. Increasing numbers of researchers have begun to study the allotropes of various materials [1,2,3,4,5,6,7,8,9,10,11,12,13,14,15,16,17,18,19,20,21,22,23,24,25,26,27,28,29,30,31,32,33,34,35,36,37,38,39,40]. According to the structure of *V* carbon [2], Li and Xing designed the structure of *P*2/*m* C_54_ and found that *P*2/*m* C_54_ is a superhard material. The mechanical anisotropy of Young’s modulus of *P*2/*m* C_54_ is slightly greater than that of the *V* carbon and diamond, while the mechanical anisotropy of Young’s modulus in the (110) plane of *P*2/*m* C_54_ is slightly smaller than that of *V* carbon. A mechanically stable and dynamically stable carbon allotrope was predicted using first-principle calculations [5], denoted as T carbon; the space group of T carbon is *Fd*-3*m*, and its space group is the same as diamond. Recently, T carbon was synthesized by Zhang et al. [6]. There are 32 carbon atoms in its conventional cell, and the center of the crystal structure has a tetrahedron, which is connected and stacked into a T carbon structure. TY carbon and Y carbon [7] with yne bonding (triple bonding) were obtained based on the T carbon and diamond structure, and the total energy of the equilibrium states of TY carbon and Y carbon are energetically more favorable than that of T carbon. TY carbon and Y carbon are approximately a half and a third as dense as T carbon, respectively. Based on a Squaroglitter structure (*t*P8 carbon), Fan et al. designed five carbon allotropes, namely, *t*P12, *t*P16, *t*P24, *o*P12, and *o*P18 carbon. All of the new structures show weaker mechanical anisotropy in Young’s modulus than that of *t*P8 carbon, while the mechanical anisotropy in Young’s modulus of all six carbon allotropes is greater than that of diamond. *t*P16 carbon is a direct band gap semiconductor material with a band gap of 1.6 eV, while other carbon structures are indirect band gap semiconductor materials. Four yne-diamonds (1-yne-diamond, 2-yne-diamond, 3-yne-diamond, and 4-yne-diamond) were proposed by Bu et al. [32], and the density of all four of the yne-diamonds was lower than that of a diamond. The 3-yne-diamond is energetically the most favorable one among the four yne-diamonds, and only the 3-yne-diamond is a direct band gap semiconductor material, with a band gap of 2.9 eV. Two *sp* + *sp*^3^ hybridized yne-diamond allotropes (called 2HYD and 4HYD, where YD is short for yne-diamond) were designed by Hu et al. [33] using first-principle calculations. Interestingly, both the electronic properties of 2HYD and 4HYD showed metallicity. Two novel carbon allotropes in *sp* + *sp*^3^ bonding networks consisting of C8 cubes, namely, Cubane-yne and Cubane-diyne [34], were dynamically and mechanically stable, and the crystal structures of cubane-yne and cubane-diyne were similar to those of supercubane [35]. Both cubane-yne and cubane-diyne show semiconductor characteristics with indirect band gaps of 3.1 eV and 2.5 eV, respectively. Recently, Costa et al. [36] proposed *n*-diamondynes using density functional theory, which enriches the carbon allotropes of the carbon–carbon single-bond family. Using the IM^2^ODE (inverse design of materials by multi-objective differential evolution) package, Zhang et al. [41] predicted five carbon allotropes with the *sp*^2^-*sp*^3^ hybridization; all the five carbon allotropes have ideal band gaps, showing that they are suitable materials for photovoltaic applications. Regarding optical properties, in the visible light range, the optical absorption coefficients of C_10_-C and C_24_-C are one order of magnitude higher than that of GaAs, C_14_-C and C_20_-D are similar to GaAs, while only C_24_-D is slightly smaller than GaAs. Due to their ideal band gaps and visible light absorption spectra, these five carbon allotropes are all potential materials for making photoelectric semiconductor devices.

In this work, the optical, mechanical, and anisotropic properties of the elastic modulus and the sound velocity of cubane-yne, cubane-diyne, supercubane, T carbon, TY carbon, Y carbon, and diamond with similar structures were studied systematically using first-principle calculations based on density functional theory (DFT) [42,43].

## 2. Methods

Structural geometric optimization calculations and physical property parameter predictions utilize density functional theory, as implemented in the Cambridge Sequential Total Energy Package (CASTEP) [44]. Vanderbilt ultrasoft pseudopotentials [45] were used, and the Broyden–Fletcher–Goldfarb–Shanno (BFGS) [46] minimization scheme was used for the structural geometric optimizations of cubane-yne, cubane-diyne, TY carbon, Y carbon, T carbon, and supercubane in this work. The exchange correlation potentials were used with the Perdew–Burke–Ernzerhof (PBE) functional of the generalized gradient approximation (GGA) [47]. A higher *k*-point separation (~0.025 Å^−1^ × 2π) [48] was used in this work; the details are 6 × 6 × 6, 4 × 4 × 4, 4 × 4 × 4, 4 × 4 × 4, 6 × 6 × 6, 8 × 8 × 8 and 12 × 12 × 12 of the conventional cell for cubane-yne, cubane-diyne, TY carbon, Y carbon, T carbon, supercubane, and diamond, respectively. In addition, a plane-wave *E*_cutoff_ energy of 520 eV was adopted for structural optimizations and physical property parameter predictions for cubane-yne, cubane-diyne, TY carbon, Y carbon, T carbon, supercubane, and diamond. The total energy convergence tests showed convergence to be within 0.001 eV/atom with the above calculation parameters. It is well known that DFT generally underestimates the electronic band gap of materials. In view of this problem, Heyd et al. proposed an easier-to-handle mixed function method, which generated HSE06 hybrid functional [49], which can be expressed as follows:

(1) $$E_{x}^{HSE}=\varepsilon E_{x}^{HF,SR}(\omega )+(1-\varepsilon )E_{x}^{PW91,SR}(\omega )+E_{x}^{PW91,LR}(\omega )+E_{c}^{PW91}$$

where the HF mixing parameter *ε* is 0.25, and the screening parameter providing good accuracy for the band gaps is *ω* = 0.207 Å^−1^ [11,49]. The electronic properties of the seven carbon allotropes are predicted uisng the PBE functional and (Heyd–Scuseria–Ernzerhof) HSE06 hybrid functional. The self-consistent convergence of the maximum ionic displacement was within 5 × 10^−4^ Å; the maximum force on the atom was 0.01 eV/Å; the total energy was 5 × 10^−6^ eV/atom; and the maximum stress was within 0.02 GPa.

## 3. Results and Discussion

**Figure 1:**
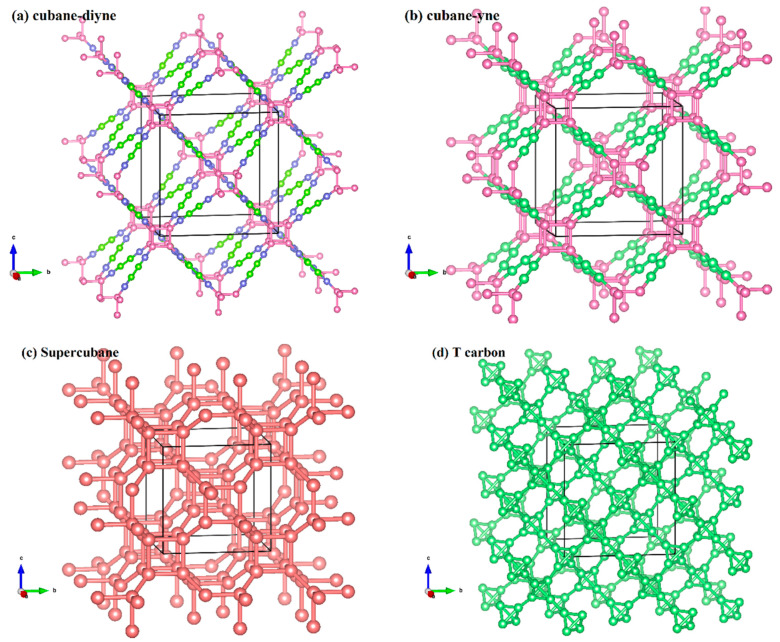

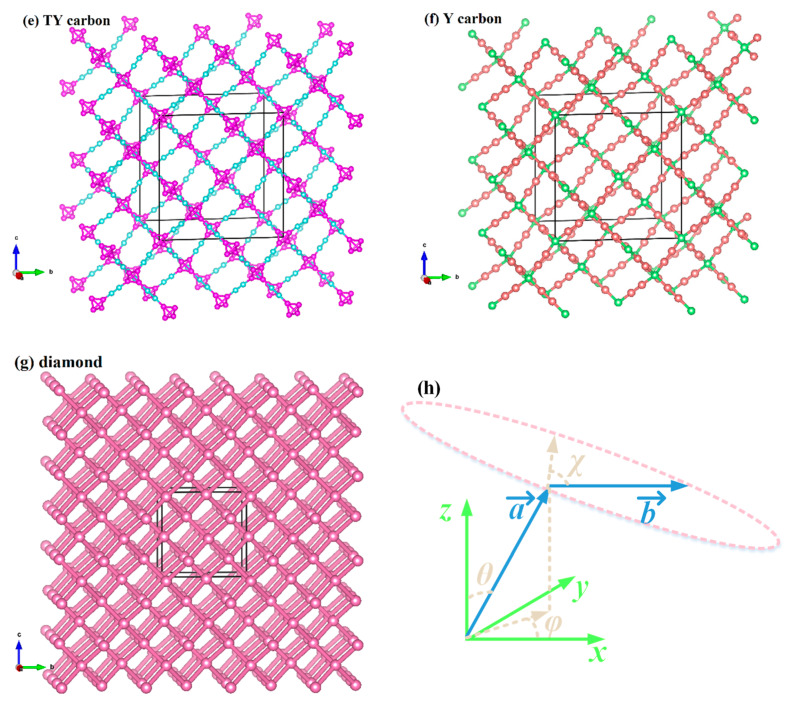
The crystal structure of cubane-diyne (**a**), cubane-diyne (**b**), supercubane (**c**), T carbon (**d**), TY carbon (**e**), Y carbon (**f**), and diamond (**g**). Definitions of angles used to describe directions in mechanical anisotropy calculations (**h**).

The crystal structures of cubane-yne, cubane-diyne, supercubane, TY carbon, Y carbon, T carbon, and diamond are similar, and their crystal structures are similar to an infinite stack of “X” letters in three-dimensional space. The crystal structures of cubane-yne, cubane-diyne, supercubane, TY carbon, Y carbon, T carbon, and diamond are shown in Figure 1a–g, respectively. The difference is that the atoms or clusters of the investigated carbon allotropes in the center of the crystal structure are different. For cubane-yne, cubane-diyne, and supercubane, there are eight atom cages in the center of the crystal structure, forming a regular hexahedron. There are four atom tetrahedron cages in the center of the crystal structure of T carbon and TY carbon. Finally, the center of the crystal structure of Y carbon and the diamond is a carbon atom. The crystal lattice parameters of the investigated carbon allotropes within the GGA level are listed in Table 1. The calculated crystal lattice parameter of diamond is 3.566 Å in this work, which is consistent with the theoretical value reported in [50]. In addition, the calculated crystal lattice parameters of diamond are in excellent agreement with the experimental value (3.567 Å) [51]. This agreement also proves that our results are reliable, so all the results in this work are based on the GGA level. From supercubane to cubane-yne, then cubane-diyne, more and more carbon atoms are hybridized by *sp*. The chain connecting the eight vertices of the regular hexahedron becomes longer, and the lattice constant increases correspondingly. Due to the larger gap in the structures, their crystal density gets smaller gradually.

The calculated elastic constants and elastic moduli (shear modulus, Young’s modulus, and bulk modulus) of the investigated carbon allotropes are shown in Table 1. All of the crystal structures of the investigated carbon allotropes in this work exhibit a cubic symmetry, with *B* = (*B*_V_ + *B*_R_)/2, *G* = (*G*_V_ + *G*_R_)/2, *B*_V_ = *B*_R_ = (*C*_11_ + 2*C*_13_)/3, *G*_V_ = (*C*_11_ − *C*_12_ + 3*C*_44_)/5, and *G*_R_ = 5 × (*C*_11_ − *C*_12_) × *C*_44_/(4*C*_44_ + 3*C*_11_ − 3*C*_12_) [52]. *C*_ij_ are the elastic constants of cubane-yne, cubane-diyne, supercubane, TY carbon, Y carbon, T carbon, and diamond. The necessary and sufficient Born mechanically stable criteria of the cubic system are taken as *C*_11_ − *C*_12_ > 0, *C*_11_ + 2*C*_12_ > 0, and *C*_44_ > 0 [53]. From the elastic constants listed in Table 1, all seven carbon allotropes satisfy the mechanically stable criteria of the cubic system, proving that cubane-yne, cubane-diyne, supercubane, TY carbon, Y carbon, T carbon, and diamond are mechanically stable. In addition, the Young’s modulus *E* is calculated using the formula *E* = 9*BG*/(3*B* + *G*). As with lattice parameters, the calculated elastic constants and elastic moduli of the investigated carbon allotropes are quite close to the theoretical and experimental values given in other references [5,7,33,34]. For supercubane, cubane-yne, and cubane-diyne, as mentioned earlier in the discussion of crystal structure differences, the carbon chain connecting the eight vertices of the regular hexahedron becomes longer, and the carbon atoms in this part of the chain are usually connected by *sp* hybridization. It is well known that the bond energy of carbon–carbon bonds with *sp* hybridization is smaller than that of carbon–carbon bonds with *sp*^3^ hybridization, so its bulk modulus from supercubane to cubane-diyne falls by 76.83%, the shear modulus is decreased by 90.26%, and Young’s modulus is decreased by 88.85%. For T carbon and diamond, the mechanical properties of T carbon are inferior to those of diamond, which results from the crystal structure. Although there are tetrahedral structures composed of carbon atoms in the crystal structure of T carbon, the number of tetrahedra is not as large as the number of tetrahedra in diamond, and the tetrahedron depends on the connection of carbon and carbon bonds in T carbon, while the crystal structure of diamond is made of tetrahedrons stacked in sequence, without the connection of other bonds. The mechanical properties of TY carbon and Y carbon are not as good as that of T carbon, as some carbon atoms adopt *sp* hybridization.

The anisotropy of crystal materials indicates that the periodicity and density of atoms are different along different directions of the crystal lattice, and this difference leads to different physical and chemical properties of crystal materials in different directions. The anisotropy of crystals is different in terms of elastic modulus, hardness, thermal conductivity, resistivity, sound velocity, electric polarization strength, etc. As an important characteristic of crystal materials, anisotropy has a very important research value. The principle of anisotropy in analytical mechanics comes from [55], and more detailed contents are described in [55]. The uniaxial stress can be expressed as a unit vector, which is advantageously described by two angles (*θ*, *φ*). We choose it as the first unit vector in the new basis set *a*. The determination of some elastic modulus (such as shear modulus and Poisson’s ratio) requires another unit vector *b*, which is perpendicular to unit vector *a*, and expressed by angle *χ*. In other words, the Young’s modulus is represented by vector *a*, while vector *b* is represented by the shear modulus and Poisson’s ratio. Their method of angle representation is shown in Figure 1h, where 0 < *θ* < π, 0 < *φ* < 2π, and 0 < *χ* < 2π. The coordinates of two vectors are

(2) $$\vec{a}=\left(\begin{array}{c} \sin\theta \cos\varphi \\ \sin\theta \sin\varphi \\ \cos\theta \end{array}\right),\underset{}{}b=\left(\begin{array}{c} \cos\theta \cos\varphi \cos\chi -\sin\varphi \sin\chi \\ \cos\theta \sin\varphi \cos\chi +\cos\varphi \sin\chi \\ -\sin\theta \cos\chi \end{array}\right)$$

The Young’s modulus is given by

(3) $$E(\theta ,\varphi )=\frac{1}{S_{11}^{\prime }}=\frac{1}{r_{1i}r_{1j}r_{1k}r_{1l}S_{ijkl}}=\frac{1}{a_{i}a_{j}a_{k}a_{l}S_{ijkl}}$$

The shear modulus is described as

(4) $$G(\theta ,\varphi ,\chi )=\frac{1}{4S_{66}^{\prime }(\theta ,\varphi ,\chi )}=\frac{1}{4r_{1i}r_{2j}r_{1k}r_{2l}S_{ijkl}}=\frac{1}{4a_{i}b_{j}a_{k}b_{l}S_{ijkl}}$$

For each *θ* and *φ*, scan angle *χ* and record the minimum, average and maximum values in this direction. The Poisson’s ratio is given by

(5) $$v(\theta ,\varphi ,\chi )=\frac{S_{12}^{\prime }(\theta ,\varphi ,\chi )}{S_{11}^{\prime }(\theta ,\varphi )}=\frac{r_{1i}r_{1j}r_{2k}r_{2l}S_{ijkl}}{r_{1i}r_{1j}r_{1k}r_{1l}S_{ijkl}}=\frac{a_{i}a_{j}b_{k}b_{l}S_{ijkl}}{a_{i}a_{j}a_{k}a_{l}S_{ijkl}}$$

**Figure 2:**
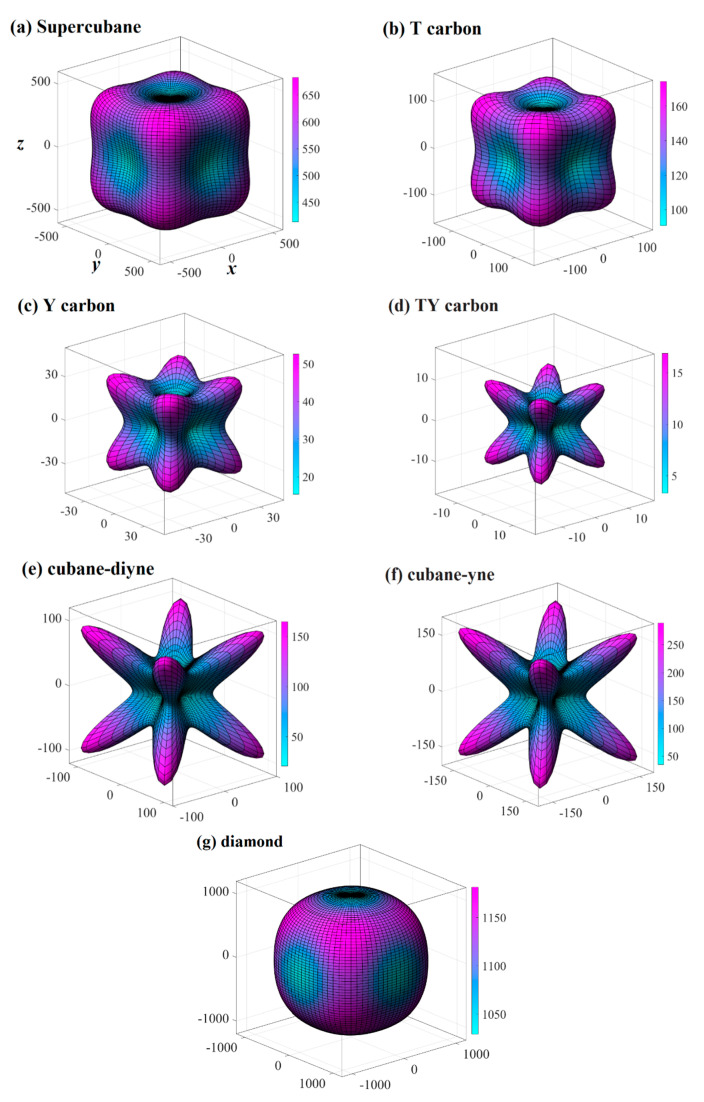
Three-dimensional contour plots of the Young’s modulus for supercubane (**a**), T carbon (**b**), Y carbon (**c**), TY carbon (**d**), cubane-diyne (**e**), cubane-diyne (**f**), and diamond (**g**).

**Figure 3:**
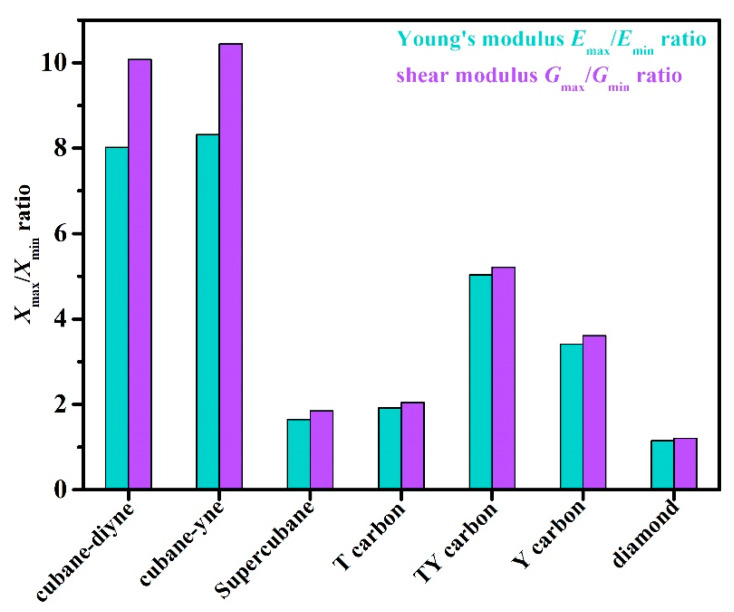
The *E*_max_/*E*_min_ ratio and *G*_max_/*G*_min_ ratio of supercubane, T carbon, Y carbon, TY carbon, cubane-diyne, cubane-diyne, and diamond.

The three-dimensional surface constructions of the Young’s modulus, shear modulus, and Poisson’s ratio of the investigated carbon allotropes are shown and investigated in this work. It can be seen from other references [56,57,58,59,60,61,62] that the three-dimensional distribution of the Young’s modulus, shear modulus, and Poisson’s ratio of isotropic crystal materials is a sphere, while the three-dimensional distribution of the Young’s modulus, shear modulus, and Poisson’s ratio of anisotropic crystal materials is not a sphere. The three-dimensional surface constructions of Young’s modulus of the investigated carbon allotropes are shown in Figure 2a–g. From Figure 2, it is obvious that all the investigated carbon allotropes exhibit elastic anisotropy in terms of the Young’s modulus as described from two angles, *θ* and *φ* (as shown in Figure 1h). Therefore, its spatial distribution is a three-dimensional figure. In addition, according to the shape of the 3D diagram of Young’s modulus in Figure 2, we can roughly distinguish the magnitude of mechanical anisotropy of Young’s modulus. In addition, from Figure 2, it can be concluded that the elastic anisotropy in the Young’s modulus of diamond is the smallest, and cubane-yne is the largest. We can use the ratio of the maximum value and the minimum value *X*_max_/*X*_min_ (*X* = *E* or *G*) to measure the elastic anisotropy in the elastic modulus. The *E*_max_/*E*_min_ ratios of the investigated carbon allotropes are shown in Figure 3. The elastic anisotropy in the Young’s modulus of Cubane-yne is the largest, while the elastic anisotropy in the Young’s modulus of diamond is the smallest; this outcome is the same as the previous prediction based on the three-dimensional distribution of the Young’s modulus. The order of elastic anisotropy in the Young’s modulus of these seven carbon materials with similar structure is diamond < supercubane < T carbon < Y carbon < TY carbon < cubane-diyne < cubane-yne. For Young’s modulus in the seven similar structures of the variability in the size of anisotropy, from the crystal structure point of view, it is because only the tetrahedron structure in diamond is stacked with each other, while the cage structure composed of four atoms or eight atoms appears in other structures. Thus, when looking out from the centre, the arrangement of atoms in all directions of the diamond is very similar, while there are cage-like structures composed of four atoms or eight atoms in other structures. The arrangement of atoms in some specific directions is similar, so the anisotropy of diamond is smaller than that of other structures. On the other hand, in the crystal structures of diamond, supercubane, and T carbon, the carbon atom only adopts *sp*^3^ hybridization, while in the other four structures (Y carbon, TY carbon, cubane-diyne, and cubane-yne), both *sp*^3^ hybridization and *sp* hybridization exist, so the anisotropy of these three structures is smaller than that of the other four carbon materials. Finally, the anisotropy of these seven similar structures is also related to the positions of carbon atoms in these crystal structures. The cubane-yne structure has three different inequivalent atom positions, and the cubane-yue has the most different inequivalent atom positions, so the mechanical anisotropy of cubane-yue is the largest. In contrast, diamond, supercabane, and T carbon only have one carbon atom inequivalent position, so their mechanical anisotropy is the smaller.

To assess the seven primary planes, i.e., (001) plane, (010) plane, (100) plane, (011) plane, (101) plane, (110) plane and (111) plane, the Young’s modulus of cubane-yne, cubane-diyne, supercubane, TY carbon, Y carbon, T carbon, and diamond are also studied in this work, and the related results are listed in Table 2. The maximum value of Young’s modulus in the (001) plane is equal to the maximum value in the (010) plane, the same as the maximum value in the (100) plane, and the minimum value of Young’s modulus is similar in these three planes. That is to say, the three primary planes have the same degree of anisotropy in Young’s modulus, which may be due to the fact that all these structures exhibit cubic symmetry, and their lattice parameters *a*, *b*, and *c* are the same. Similar to the (001), (010), and (100) planes, for the (011), (110), and (101) planes the maximum value of Young’s modulus is the same, as is the minimum. The maximum values of the Young’s modulus for the investigated carbon allotropes are equal to the minimum values in the (111) plane; therefore, the Young’s modulus of the investigated carbon allotropes in the (111) plane reveals elastic isotropy. From Table 2, the elastic anisotropy of the Young’s modulus in the (011) plane, (110) plane, and (101) plane are greater than that of the (001) plane, (010) plane, and (100) plane. The order of elastic anisotropy in the Young’s modulus of these seven main planes of the investigated carbon allotropes is (111) plane < (001) plane = (010) plane = (100) plane < (011) plane = (110) plane = (101) plane.

**Figure 4:**
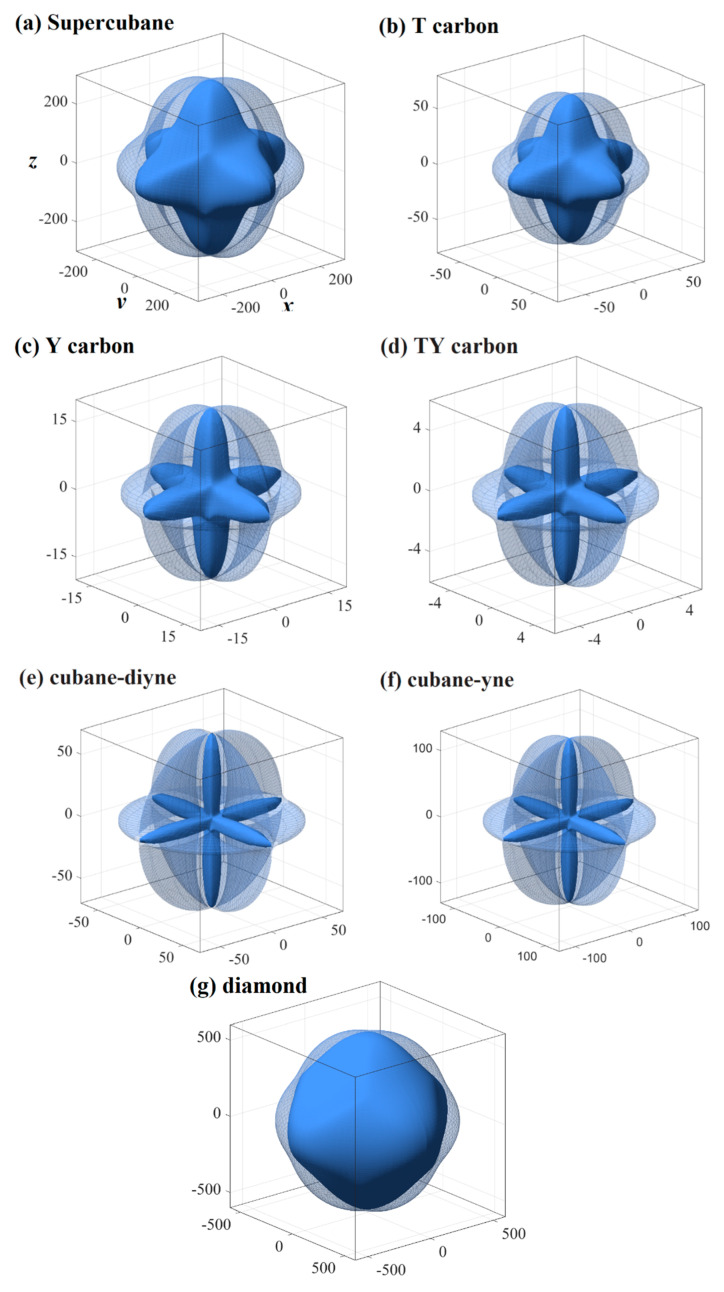
Three-dimensional contour plots of the shear modulus for supercubane (**a**), T carbon (**b**), Y carbon (**c**), TY carbon (**d**), cubane-diyne (**e**), cubane-diyne (**f**), and diamond (**g**).

**Figure 5:**
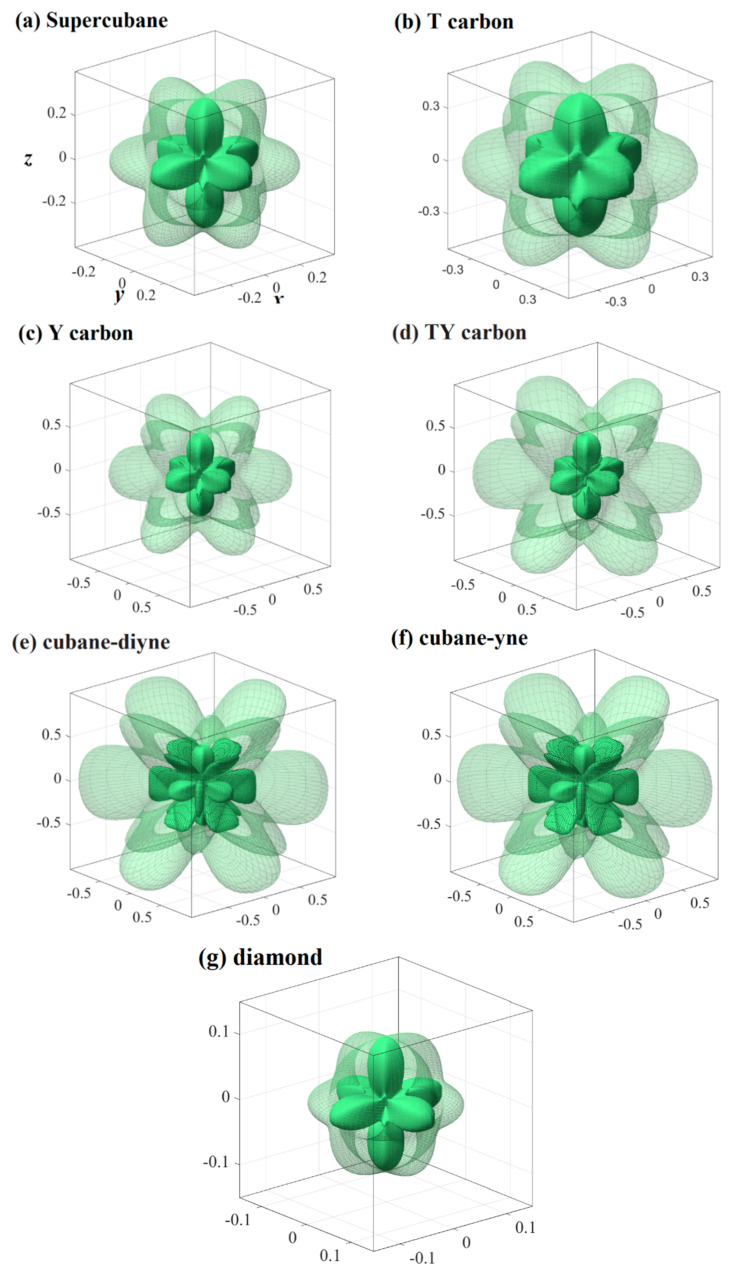
Three-dimensional contour plots of the Poisson’s ratio for supercubane (**a**), T carbon (**b**), Y carbon (**c**), TY carbon (**d**), cubane-diyne (**e**), cubane-diyne (**f**), and diamond (**g**).

The three-dimensional surface constructions of the shear modulus and Poisson’s ratio of the investigated carbon allotropes are shown in Figure 4 and Figure 5, respectively. As shown in Figure 1h, because the shear modulus is described from three angles *θ*, *φ* and *χ*, and the scanning angle *χ* is added, its spatial distribution should be a four-dimensional figure. With the scanning angle *χ*, we can make three-dimensional figures of the maximum and minimum values of shear modulus and Poisson’s ratio. Similar to Young’s modulus, according to the shape of the 3D diagram of shear modulus and Poisson’s ratio in Figure 4 and Figure 5, we can roughly distinguish the magnitude of mechanical anisotropy of shear modulus and Poisson’s ratio. In Figure 4 and Figure 5, the surface composed of dotted lines is the set of maximum shear modulus and Poisson’s ratio, while the surface composed of solid lines is the set of minimum shear modulus and Poisson’s ratio. From Figure 4, the minimum value of the shear modulus is close to the centre of the three-dimensional distribution, that is, the minimum value of the shear modulus decreases gradually, indicating that the elastic anisotropy of the shear modulus increases gradually. The maximum and the minimum values of the shear modulus and Poisson’s ratio of the investigated carbon allotropes in the (001) plane, (010) plane, (100) plane, (011) plane, (101) plane, (110) plane, and (111) plane are also listed in Table 2. All of the maximum and the minimum values of the shear modulus and Poisson’s ratio of the investigated carbon allotropes in these seven main planes are the same. This is mainly due to the fact that all seven carbon materials exhibit cubic symmetry, which is the most symmetrical among the seven symmetries. Similar to Young’s modulus, the *G*_max_/*G*_min_ ratios of the investigated carbon allotropes are shown in Figure 3. As the minimum value of the Poisson ratio of some carbon materials is 0, it is not suitable to use the *v*_max_/*v*_min_ ratio to measure the Poisson ratio. Instead, the difference between the maximum value and the minimum value of the Poisson ratio is used to measure the elastic anisotropy. From Figure 3, the order of elastic anisotropy in the shear modulus and Poisson’s ratio of these seven carbon materials with similar structure is diamond (*v*_max_ − *v*_min_ = 0.10) < supercubane (0.42) < T carbon (0.56) < Y carbon (1.00) < TY carbon (1.23) < cubane-diyne (1.33) < cubane-yne (1.35). The size of anisotropy is greatly related to its own crystal structure, the constituent elements and the crystal system to which it belongs. The seven materials studied in this work are only composed of carbon elements and belong to the cubic crystal system, so the anisotropy magnitude of shear modulus and Poisson’s ratio is similar to Young’s modulus as discussed earlier, which is related to its own crystal structure.

**Figure 6:**
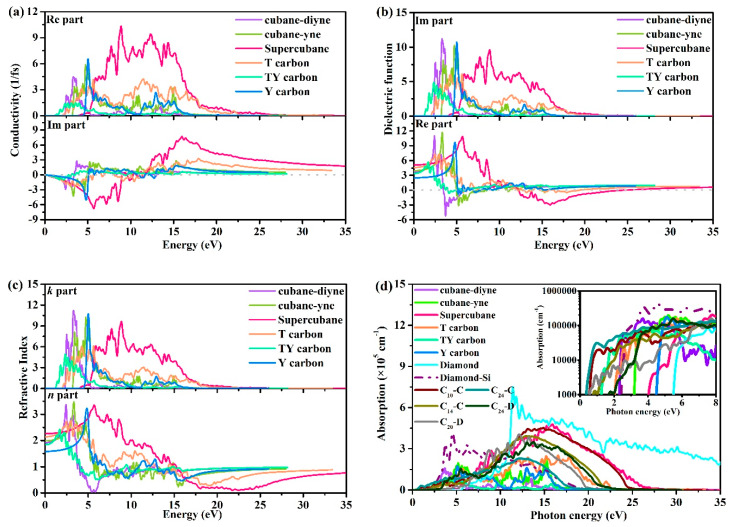

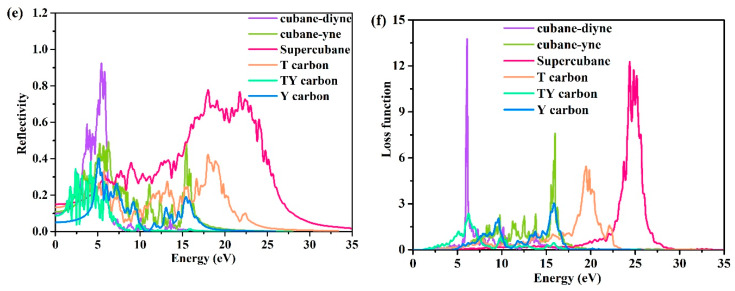
Calculated optical properties for the investigated carbon allotropes: Conductivity (**a**), dielectric function (**b**), refractive index (**c**), absorption coefficient (**d**), optical reflectivity spectrum (**e**) and loss function (**f**).

Optical properties play an important role in our understanding of material properties. For example, dielectric function is the key optical quantity to extract optical properties such as the absorption spectrum, energy loss function, refractive index, and reflectivity. In this paper, the conductivity, dielectric function, refractive index, absorption, reflectivity, and loss function of cubane-yne, cubane-diyne, supercubane, TY carbon, Y carbon, and T carbon are studied, and the results are shown in Figure 6. The imaginary part and the real part of the conductivity of the investigated carbon allotropes are shown in Figure 6a. In the real part of the conductivity, when the photon energy exceeds 2.202, 3.049, 3.640, 1.780, 0.996, and 4.199 1/fs, the conductivity is not zero. The conductivity of Y carbon starts late and ends early, which means that it occupies a small energy range of photons. The order of the region size of the photon energy range corresponding to the region with conductivity not equal to 0 is T carbon > supercubane > TY carbon > cubane-yne > cubane-diyne > Y carbon.

As a bridge between the microphysical process and solid electronic structure, the dielectric function reflects the band structure and other optical information of solid materials. The imaginary part and the real part of the dielectric functions of cubane-yne, cubane-diyne, supercubane, TY carbon, Y carbon, and T carbon are shown in Figure 6b. The calculated static dielectric constants are 3.666, 3.938, 5.118, 4.590, 3.380, and 2.502 for cubane-diyne, cubane-yne, supercubane, T carbon, TY carbon, and Y carbon, respectively. The curve of the imaginary part of the dielectric function gives the threshold value of the direct optical transition between the highest valence band and the lowest conduction band, which is the basic absorption edge. In the imaginary part, when the energies of photons are 2.202, 3.049, 3.640, 1.780, 0.996, and 4.199 for cubane-diyne, cubane-yne, supercubane, T carbon, TY carbon, and Y carbon, the imaginary part is not zero. When the photon energy is more than 5 eV and less than 20 eV, the peak areas of the imaginary parts of the dielectric functions of the investigated carbon allotropes mainly appear in this region. The imaginary part of the dielectric function of supercubane has a higher dielectric function than those of the other five carbon allotropes.

The refractive index *n* and extinction coefficient *k* of cubane-diyne, cubane-yne, supercubane, T carbon, TY carbon, and Y carbon are shown Figure 6c. At ambient pressure, the static refractive indices *n*(0) are 1.9, 2.0, 2.3, 2.1, 1.8, and 1.6 for cubane-diyne, cubane-yne, supercubane, T carbon, TY carbon, and Y carbon, respectively. The *n*(*ω*) values of cubane-diyne, cubane-yne, supercubane, T carbon, TY carbon, and Y carbon increase with increasing photon energy from 0 to 5 eV and reach peaks at approximately 3.4, 3.5, 3.4, 2.7, 2.4, and 3.3 eV for cubane-diyne, cubane-yne, supercubane, T carbon, TY carbon, and Y carbon, respectively. The absorption coefficient defines how much energy a material absorbs. The absorption coefficients of the seven carbon allotropes, diamond-Si, and C_10_-C, C_14_-C, C_20_-D, C_24_-C, C_24_-D [41] are shown in Figure 6d. It is noted that all the absorption coefficients of cubane-diyne, cubane-yne, supercubane, T carbon, TY carbon, and Y carbon, C_10_-C, C_14_-C, C_20_-D, C_24_-C, C_24_-D, and diamond are smaller than that of diamond-Si in the visible light area (1.6–3.2 eV). When the photon energy is 3.6, 4.2, and 5.3 eV, the absorption coefficients of supercubane, Y carbon, and diamond are not zero. In other words, the three materials will not absorb visible light. Although the absorption of T carbon (1.8 eV) and TY carbon (1.0 eV) to visible light occurs earlier than that of diamond-Si (2.1 eV), their absorption coefficient is less than that of diamond-Si, which is approximately half that of diamond-Si. The TY carbon is the first one to absorb photons mentioned in this work among the seven carbon allotropes. However, compared with C_10_-C, C_14_-C, C_20_-D, C_24_-C, and C_24_-D, these five carbon allotropes begin to absorb the energy of photons earlier than TY carbon. Among the five new structures proposed by Zhang et al. [41], C_24_-D is the latest material to start to absorb photons at 0.4 eV. Among the known single junction solar cell absorbers, GaAs has the highest light absorption efficiency [41], and in the visible light range, the optical absorption coefficients of C_10_-C and C_24_-C are one order of magnitude higher than that of GaAs. Using the same mapping method as in reference [41], we compared the absorption spectra of seven carbon allotropes in the visible region and five carbon materials proposed by Zhang et al. [41]. The related results are shown in the inset of Figure 6d. From the inset of Figure 6d, TY carbon is similar to that of C_10_-C and C_20_-C in the visible light range, while T carbon, cubane-diyne, and diamond-Si are similar to that of C_14_-C and C_20_-D in the visible light range. Among the materials that can absorb visible light in the visible light area, C_24_-D still has the worst visible light absorption ability. Cubane-yne just absorbs photons in the visible light area, and other materials do not absorb photons in the visible light area. Therefore, TY carbon, T carbon, and cubane-diyne may also be used as potential semiconductor materials for photoelectric applications.

The reflective coefficients of the investigated carbon allotropes are shown in Figure 6e. The peaks occur at approximately 0.923, 0.493, 0.778, 0.424, 0.385, and 0.402 for cubane-diyne, cubane-yne, supercubane, T carbon, TY carbon, and Y carbon, respectively. The loss function describes the energy loss of electrons in the process of fast passage through crystal materials. The loss functions of the investigated carbon allotropes are shown in Figure 6f. It can be noted that the positions of the loss functions of the investigated carbon allotropes are different, and the peak values are also different. Cubane-diyne lies mainly between 5 and 10 eV, cubane-yne lies mainly between 5 and 20 eV, TY carbon lies mainly between 2 and 8 eV, Y carbon lies mainly between 2 to 12 eV and 12 to 17.5 eV, T carbon lies mainly between 15 and 23 eV, and supercubane lies between 20 and 28 eV. The peak magnitude of TY carbon is the smallest, and the cubane-diyne magnitude is the greatest.

By using the semi-empirical formula of the elastic constant of the Debye temperature [63,64], we calculated the Debye temperature of these seven carbon materials with similar structures. The semi-empirical formula is *Θ*_D_ = *v*_m_(*h*/*k*_B_)[3*n*/(4*π*)(*N*_A_*ρ*/*M*)]^1/3^, where *v*_m_ = [(2/$v_{s}^{3}$ + 1/$v_{p}^{3}$)/3]^−1/3^, *v*_s_ = (*G*/*ρ*)^1/2^, *v*_p_ = [(*B* + 4*G*/3)/*ρ*]^1/2^, *n* is the number of atoms in the molecule, *M* is the molecular weight, *ρ* is the crystal density, *k*_B_ is Boltzmann’s constant, *h* is Planck’s constant, *N*_A_ is Avogadro’s number, *v*_s_ is the shear sound velocity, *v*_p_ is the compressional sound velocity, *v*_m_ is the mean sound velocity, and the *Θ*_D_ is Debye temperature. In addition, the crystal densities of the investigated carbon allotropes are listed in Table 1. The compressional sound velocity, shear sound velocity, mean sound velocity, and Debye temperatures of cubane-yne, cubane-diyne, supercubane, TY carbon, Y carbon, T carbon, and diamond are listed in Table 3. The calculated Debye temperature of diamond is 2226.32 K, which is in excellent agreement with the theoretical values previously reported at 2230 K [65] and 2220 K [66]. The order of Debye temperatures of these seven carbon materials with similar structures is diamond > supercubane > cubane-yne > T carbon > cubane-diyne > Y carbon > TY carbon. According to the semi empirical formula of Debye temperature, it is related to the shear modulus and bulk modulus, and the order of Debye temperature is the same as that of their shear modulus, that is, the influence of shear modulus on Debye temperature is greater than that of the bulk modulus.

The shear sound velocity and compressional sound velocity are also anisotropic. Since all the cubane-yne, cubane-diyne, supercubane, TY carbon, Y carbon, T carbon, and diamond samples are cubic crystal systems, the calculation formulas of the sound velocity in the three propagation directions of [100], [110] and [111] are [65,67]: [100] *v*_p_ = (C11/ρ)1/2, [010] *v*_s1_ = [001] *v*_s2_ = (C44/ρ)1/2, [110] *v*_p_ = [(*C*_11_ + *C*_12_ + 2*C*_44_)/2*ρ*]^1/2^, [1–10] *v*_s1_ = [(*C*_11_ − *C*_12_)/*ρ*]^1/2^, [111] *v*_p_ = [(*C*_11_ + 2*C*_12_ + 4*C*_44_)/3*ρ*]^1/2^, and [11–2] *v*_s1_ = *v*_s2_ = [(*C*_11_ − *C*_12_ + *C*_44_)/3*ρ*]^1/2^, respectively. The shear sound velocity and compressional sound velocity of cubane-yne, cubane-diyne, supercubane, TY carbon, Y carbon, T carbon, and diamond along the [100], [110], and [111] directions are also listed in Table 3. From Table 3, it is seen that the shear sound velocity and compressional sound velocity of the investigated carbon allotropes differ along the [100], [110], and [111] propagation directions. Among the [100], [110], and [111] propagation directions, in the [110] propagation direction, the anisotropic ratio of sound velocity [(the maximum magnitude of sound velocity—the minimum magnitude of sound velocity)/the maximum magnitude of sound velocity] of TY carbon is the largest, and the anisotropic ratio of sound velocity of cubane-diyne in the [100] propagation direction is the smallest. In addition, the anisotropic ratio of the sound velocity of both TY carbon and Y carbon on the three propagation directions exceeds 50%.

**Figure 7:**
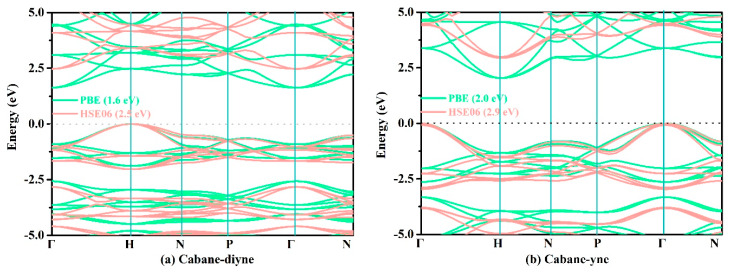

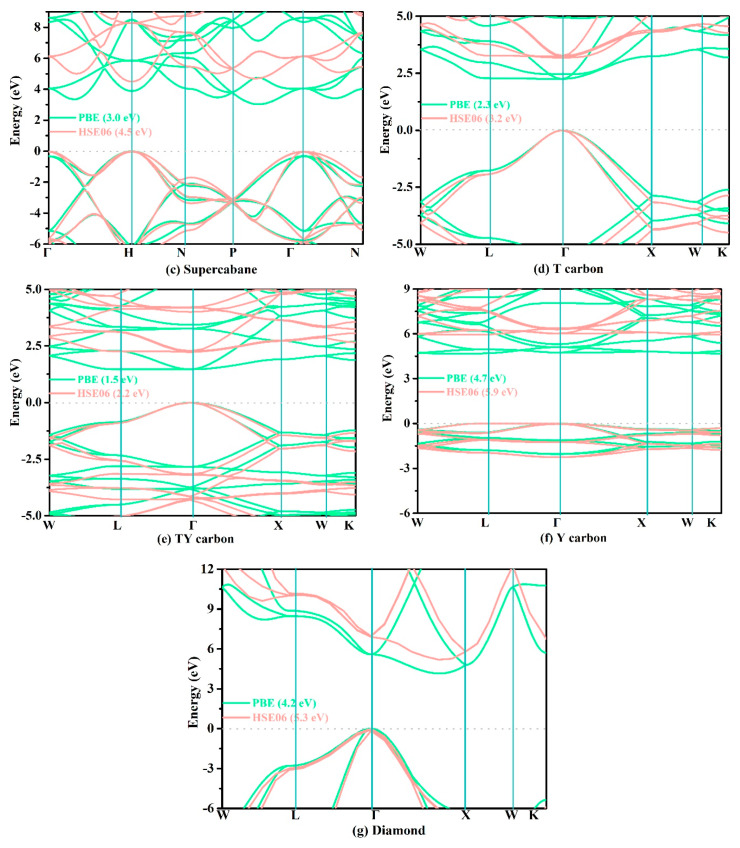
The calculated band structures of cubane-diyne (**a**), cubane-yne (**b**), supercubane (**c**), T carbon (**d**), TY carbon (**e**), Y carbon (**f**), and diamond (**g**), respectively.

Finally, we used the PBE functional and HSE06 hybrid functional to predict the electronic band structures of these structures; the relevant results are shown in Figure 7. As shown in Figure 7, the coordinates of the high symmetry points for cubane-diyne, cubane-yne, and supercubane are Γ (0.000, 0.000, 0.000) → H (0.500, −0.500, 0.500) → N (0.000, 0.000, 0.500) → P (0.250, 0.250, 0.250) → Γ (0.000, 0.000, 0.000) → N (0.000, 0.000, 0.500). The coordinates of the high symmetry points for T carbon, TY carbon, Y carbon, and diamond are W (0.500, 0.250, 0.750) → L (0.500, 0.500, 0.500) → Γ (0.000, 0.000, 0.000) → X (0.500, 0.000, 0.500) → W (0.500, 0.250, 0.750) → K (0.375, 0.375, 0.750). From Figure 7, the band gaps of cubane-yne, cubane-diyne, and supercubane accord well with those from previous reports (2.5 eV for cubane-diyne [34], 3.1 eV for cubane-yne [34], 4.2 eV for supercubane [34]) for the HSE06 hybrid functional. For TY carbon, Y carbon, T carbon, and diamond, the band gaps using the PBE function also accord well with previous reports (2.2 eV for T carbon [7], 4.7 eV for Y carbon [7], 1.5 eV for TY carbon [7], 4.1 eV for diamond [7], 4.1 eV for diamond [68]). As mentioned above, the results obtained using two different methods (PBE functional and HSE06 hybrid functional) prove that the electronic structures and band gaps obtained using the PBE and HSE06 functional are reliable. For the experimental band gap (5.5 eV [62], 5.5 eV [69]) of diamond, the band gap predicted using the HSE06 hybrid functional is 5.3 eV, which is in excellent agreement with the experimental values. It is well known that DFT generally underestimates the electronic band gap of materials by about 30%–50% [62]. As for the underestimation of the band gap, for cubane-diyne it is underestimated by 51.20% with the PBE functional compared to the HSE06 hybrid functional, which was slightly smaller than that of TY carbon (51.98%). The diamond, which is the one with the least underestimated band gap value, is only less than the true value by 24.90%. It is well known that diamond is an indirect band gap semiconductor. In our work, diamond, cubane-diyne, cubane-yne, and Y carbon have indirect band gaps under both PBE and HSE06 levels, while the TY carbon and T carbon are direct band gap semiconductor materials. When the PBE and HSE06 hybrid functional are used to estimate the band structure and band gap of supercubane, all the calculation parameters are the same as those of cubane-diyne, cubane-yne, T carbon, TY carbon, Y carbon, and diamond. In addition, supercubane is an indirect band gap under the PBE functional, while it is a direct band gap under the HSE06 hybrid functional, which is not consistent with a previous report [34]. It is possible that there were some differences among the parameters used in these two works.

## 4. Conclusions

Based on density functional theory, seven carbon materials with X-type structures—cubane-yne, cubane-diyne, supercubane, TY carbon, Y carbon, T carbon, and diamond—are investigated in this work to assess their optical and mechanical properties; anisotropy in the Young’s modulus, shear modulus, and Poisson’s ratio; and sound velocity. First, all seven similar structures are mechanically stable. Second, related to its own crystal structure, the constituent elements and the crystal system to which it belongs, cubane-yne exhibits the largest elastic anisotropy in the Young’s modulus, shear modulus, and Poisson’s ratio, while the elastic anisotropy in the Young’s modulus, shear modulus and Poisson’s ratio of diamond is the smallest. Among the [100], [110], and [111] propagation directions, in the [110] propagation direction, the anisotropic ratio of the sound velocity of TY carbon is the largest, and the anisotropic ratio of the sound velocity of cubane-diyne in the [100] propagation direction is the smallest. Third, the cubane-diyne, cubane-yne, and Y carbon are indirect band gap semiconductor materials, while the TY carbon, supercubane, and T carbon are direct band gap semiconductor materials with the HSE06 hybrid functional. Our research hopes to play a positive role in promoting the development of carbon materials science. Finally, among the known single junction solar cell absorbers, GaAs has the highest light absorption efficiency; the optical absorption coefficients of C_10_-C, C_24_-C, and TY carbon are one order of magnitude higher than that of GaAs in the visible light range; while T carbon, cubane-diyne, diamond-Si, C_14_-C, and C_20_-D are similar to that of GaAs in the visible light range. Therefore, TY carbon, T carbon, and cubane-diyne may also be used as potential semiconductor materials for photoelectric applications.

## Figures and Tables

**Table 1:** The lattice parameters (Å), volumes of the conventional cell (Å^3^), elastic constants (GPa), and elastic moduli (GPa) of the investigated carbon allotropes.

	*a*	*V*	*ρ*	*C*_11_	*C*_12_	*C*_44_	*B*	*G*	*E*
Cubane-yne	7.845 ^a^	15.088	1.322	151.5	128.4	119.3	136.1	50.7	136.0
	7.837 ^b^	15.052	1.326				148		
Cubane-diyne	10.815 ^a^	26.355	0.757	80.2	75.1	63.3	76.8	22.5	62.7
	10.811 ^b^	26.345	0.758				84.6		
TY carbon	13.441 ^a^	34.943	0.526	55.1	52.9	5.9	53.6	3.1	8.8
	13.460 ^c^		0.523				54.2		
Y carbon	9.621 ^a^	22.264	0.896	89.4	78.9	18.9	82.4	11.4	31.6
	9.636 ^c^		0.894				82.9		
T carbon	7.501 ^a^	13.189	1.512	200.9	136.3	66.3	157.8	49.7	135.7
	7.520 ^d^		1.503				159		
Supercubane	4.813 ^a^	6.966	2.863	544.7	224.9	295.4	331.5	230.9	562.5
	4.853 ^b^	7.148	2.792				329		
Diamond	3.566 ^a,e^	11.341					431	522	1116
	3.567 ^f^	11.346					442^g^		

^a^ This work, ^b^ [34], ^c^ [7], ^d^ [5], ^e^ [50], ^f^ [51]—experimental, ^g^ [54]—experimental.

**Table 2:** The calculated maximum (GPa), minimum values (GPa), and *X*_max_/*X*_min_ ratio (*X*= *G* or *E*) of cubane-diyne, cubane-diyne, supercubane, T carbon, TY carbon, diamond, and Y carbon.

		*E*_max_	*E*_min_	Ratio	*G*_max_	*G*_min_	Ratio	*v*_max_	*v*_min_
(100) plane (010) plane (001) plane	Cubane-yne	102.03	34.68	2.94	124.06	11.88	10.44	1.35	0.00
	Cubane-diyne	59.87	20.55	2.91	71.04	7.05	10.08	1.33	0.00
	TY carbon	8.43	3.36	2.51	5.87	1.13	5.19	1.23	0.00
	Y carbon	32.84	15.40	2.13	18.94	5.24	3.61	1.00	0.00
	T carbon	141.76	90.67	1.56	66.33	32.28	2.05	0.63	0.07
	Supercubane	587.30	413.24	1.42	295.39	159.89	1.85	0.42	0.00
	Diamond	1139.60	1029.42	1.11	566.46	467.07	1.21	0.11	0.01
(011) plane (101) plane (110) plane	Cubane-yne	289.36	34.68	8.34	124.06	11.88	10.44	1.35	0.00
	Cubane-diyne	165.22	20.55	8.04	71.04	7.05	10.08	1.33	0.00
	TY carbon	17.00	3.36	5.06	5.87	1.13	5.19	1.23	0.00
	Y carbon	52.77	15.40	3.43	18.94	5.24	3.61	1.00	0.00
	T carbon	174.54	90.67	1.93	66.33	32.28	2.05	0.63	0.07
	Supercubane	683.24	413.24	1.65	295.39	159.89	1.85	0.42	0.00
	Diamond	1181.76	1029.42	1.15	566.46	467.07	1.21	0.11	0.01
(111) plane	Cubane-yne	102.03	102.03	1.00	124.06	11.88	10.44	1.35	0.00
	Cubane-diyne	59.87	59.87	1.00	71.04	7.05	10.08	1.33	0.00
	TY carbon	8.43	8.43	1.00	5.87	1.13	5.19	1.23	0.00
	Y carbon	32.84	32.84	1.00	18.94	5.24	3.61	1.00	0.00
	T carbon	141.76	141.76	1.00	66.33	32.28	2.05	0.63	0.07
	Supercubane	587.30	587.30	1.00	295.39	159.89	1.85	0.42	0.00
	Diamond	1139.60	1139.60	1.00	566.46	467.07	1.21	0.11	0.01

**Table 3:** The compressional sound wave velocity *v_s_*, shear sound wave velocity *v_p_* and mean sound wave velocity *v_m_* (m/s), and the Debye temperature (K) in the seven similar structures.

	Cubane-yne	Cubane-diyne	TY Carbon	Y Carbon	T Carbon	Supercubane	Diamond
[100]:[100]*v_p_*	10,705	10,293	10,235	9989	11,527	13,793	17,305
[100]:[010]*v_s_*_1_	9500	9144	3349	4593	6622	10,158	12,688
[100]:[001]*v_s_*_2_	9500	9144	3349	4593	6622	10,158	12,688
[110]:[110]*v_p_*	14,004	11,897	11,185	10,724	14,114	15,414	18,103
[110]:[1–10]*v_s_*_1_	4180	2596	2045	3423	6536	10,569	16,292
[110]:[001]*v_s_*_2_	9855	9960	10,028	9384	9494	8863	5832
[111]:[111]*v_p_*	14,942	14,593	10,813	10,959	12,761	15,917	18,362
[111]:[1–12]*v_s_*_1_	5992	5488	2265	3307	5372	8463	11,993
[111]:[1–12]*v_s_*_2_	5992	5488	2265	3307	5372	8463	11,993
*v_p_*	12,414	11,880	10,480	10,438	12,173	15,133	17,922
*v_s_*	6193	5453	2428	3567	5733	9094	12,207
*v_m_*	6948	6144	2774	4057	6452	10,059	13,307
*Θ_D_*	838.22	615.46	246.10	429.86	814.04	1556.87	2224.84
